# Facility Layout Planning with SHELL and Fuzzy AHP Method Based on Human Reliability for Operating Theatre

**DOI:** 10.1155/2019/8563528

**Published:** 2019-01-14

**Authors:** QingLian Lin, Duojin Wang

**Affiliations:** ^1^School of Management, Xiamen University, Xiamen 361005, China; ^2^School of Medical Instrument and Food Engineering, University of Shanghai for Science and Technology, Shanghai 200093, China

## Abstract

A well-design facility layout planning refers to the reduction of the operation cost in the manufacturing and service industry. This work consists of reliability analysis of facility layout for an operating theatre; it aims at proposing a new evaluation approach, which integrated the fuzzy analytic hierarchy process and human reliability tool, for optimization of facility layout design with safety and human factors in an operating theatre. Firstly, the systematic layout planning is used to design the layout schemes on the basis of field investigations. Then, the criteria system is proposed based on human reliability analysis from four perspectives: software, hardware, environment, and liveware. Finally, the fuzzy analytic hierarchy process, a fuzzy extension of the multicriteria decision-making technique analytic hierarchy process, is used to compare these layout schemes based on the criteria system. The results that are obtained reveal interesting properties of facility layout planning in hospitals. It reveals that decision in selecting a suitable layout must meet not only the strategies and goals of the system but also meet the safety, security, and reliability of the system.

## 1. Introduction

With the large aging populations, restricted health care resource, and rising expectations, the effective organization and provision of health care have become more important and complex [1]. The health care industry has faced a significant change, a worldwide demographic change. The world must actively respond to the new challenges in demographic development, with fundamental changes in the existing structures in all areas of society [2]. Hospital, as a health care institution, is also facing the challenge to increase efficiency for an increasingly diverse situation [3]. It is critical to take strategic steps for optimizing the structure, strategies, and systems of hospitals, as well as the allocation of resources among different hospitals or within a hospital [4, 5]. The administrators of hospitals are seeking new ways to improve the efficiency and effectiveness, especially the utilization of health care resources. And hospital management needs to control the health cost and improve financial assets simultaneously [6]. The operating theatres, as the key resource in a hospital, present the largest cost source and act as the bottleneck which would influence the quality and efficiency of the overall health care service [7]. For the productive and effective functions of hospitals, management should ensure an optimum functioning of the system components. Hospital administrators tend to focus on the productivity, safety, and quality of providing medical treatment, thereby introducing some optimization tools that can be employed to improve the performance of hospitals and to increase the patients' satisfaction, but the poor or partial integration of those industrial layout design tools has been adopted to increase the process efficiency in hospitals.

Facility layout design determines the relative location of departments and machines within a plant and aims at obtaining the most effective facility arrangement and minimizing the material handling costs [8]. A good layout design must meet a set of criteria and objectives, e.g., area requirements, cost, communication, and safety [9]. There is a great quantity of research about designing and optimizing facilities layout in manufacturing and service systems [10–13]. However, a small quantity of research studies the facility layout in health care service. In health care systems, the design of the facility layout has been associated with health care quality, work efficiency, and cost-effectiveness [14]. Hendrich et al. [15] identified that the nursing work process and nursing unit design have an effect on the efficiency of nursing care and the safe delivery of care; they observed the process in 36 US hospitals and found that nurses spent 19.3% of their time on patient care activities, and the median walking distance covered by a nurse on dayshift was 3 miles. Lin et al. [16] proposed an approach to design and optimize the facility layout for operating rooms using the systematic layout planning and fuzzy constraint theory. However, they just considered “high logistics efficiency” and “high space utilization rate” as objectives for evaluation. It needs a more scientific approach to create a criteria system to assess the layout plans. Wang et al. [17] explored an approach that integrated the lean principles and simulation optimization to analyze the hospital emergency department layout design and staff assignment problems; they found that the linear layout was better than a U-shaped layout in both waiting time and service level. Chraibi et al. [18] proposed a mixed integer linear programming formulation considering the size, the orientation, and the shape of rooms for the operating theatre layout problems with the two objectives: minimize the interdepartmental traveling costs and maximize the closeness of the facilities. And Arnolds and Nickel [19] proposed a multiperiod layout planning for hospital wards; they developed five mathematical models to generate ward layouts according to the varying demand for different sized bedrooms in multiple periods. Some research has shown that the health outcomes can be improved through good hospital design [20].

In the literature, most research focused on hospital settings and design principles for primary care. But there are limited numbers of literature studies related to the facility layout optimization in operating theatres, considering about the security factors, especially the safety problem caused by the use of people. Therefore, this work tried to introduce the human reliability in facility layout planning in an operating theatre. Human reliability assessment is an assessment of methods and models that is used to predict the occurrence of human errors. It is a useful approach that have been used in high risk industries systems, such as nuclear and aerospace, to prevent accidents [21, 22]. Numerous research studies related on human reliability have been carried out in industrial systems safety, but few in health care systems. For instance, Jang et al. [23] studied a new framework of human reliability analysis to evaluate soft control execution error in advanced main control rooms and finally developed a nominal human error probabilities and recovery failure probability database. Mohammadfam et al. [24] compared two human error analysis methods, SPAR-H and CREAM, based on multicriteria decision-making AHP in quantifying a human error in nursing practice. Su et al. [25] proposed a dependence assessment approach based on the Dempster–Shafer evidence theory and AHP to handle dependence on HRA; they concluded that the DSET with AHP can improve the flexibility and reduce the subjectivity to dependently assess human task in HRA. Lin et al. [26] explored an assessment model based on qualitative and quantitative technologies for human reliability of medical devices to improve the safety. Moraru et al. [27] developed and applied a HRA model for mine dispatchers and analyzed the importance of HRA in system safety; they concluded that human reliability assessment could reduce the risk in high technology production systems. Human reliability analysis is growing in popularity among safety analysis in manufacturing/service systems. Nevertheless, there are seldom studies about human reliability applied to optimize the facility layout planning in health care systems.

The assessment information in human reliability for health care systems always involves uncertain, incomplete, and imprecise information. It is difficult to obtain or lack data management in healthcare systems. Fuzzy AHP is a structured technique for solving complex multicriteria decision-making problems based on mathematics and psychology; it is able to process uncertain variables. Consequently, fuzzy AHP was used in this work to develop an assessment model for facility layout planning in an operating theatre. There are numerous research works on Fuzzy AHP in the literature. For instance, Torabi-Kaveh et al. [28] developed a multicriteria decision analysis process for determining suitable sites for landfill construction and combining geographic information system analysis with a fuzzy AHP. Wang et al. [29] used a fuzzy AHP to select candidate transport modes by fuzzy pair-wise comparison matrices and last found an optimal transport mode for Kinmen military. Tyagi et al. [30] applied fuzzy AHP to improve the supply chain performance for automobile industries by examining the preferable alternative. Wang et al. [31] proposed a two-stage fuzzy AHP model to deal with the qualitative nature and the uncertainty parameters in the decision-making process for risk assessment in the fashion supply chain. And Sangwan and Kodali [32] integrated AHP and fuzzy logic to deal with the vague and imprecise data and weigh the different factors between each pair; they concluded that the proposed model can minimize the cost function and maximize the closeness function for assignment of facilities. Whilst fuzzy AHP in multicriteria decision-making in industries has been well-accepted, the application of fuzzy AHP to make a decision based on the criteria system by human reliability analysis in health care systems is rare.

This paper attempts to design and optimize the layout plan for operating theatres, using the systematic layout planning (SLP) to design the layout plans with human reliability analysis (HRA) and fuzzy analytic hierarchy process (AHP) to develop the criteria system and assess the layout plans to select the best one.

The purposes of this paper are as follows:

- Briefly outline the complexity of the facility layout in the operating theatre
- Discuss the design process and how it is applied in an operating theatre
- Briefly review the literature on systematic facility layout design in health care systems and on HRA and fuzzy AHP
- Develop a theoretical methodology for design and optimization of the operating theatre layout
- Present a case study that evaluates the application of systematical facility planning for designing layout plans and HRA and fuzzy AHP for assessment and select the best plan for an operating theatre
- Extend the study to show how the proposed methods contribute to the other fields

## 2. Materials and Methods

### 2.1. Systematic Layout Planning

Systematic layout planning (SLP) is a procedural layout design approach which is used to arrange a workplace in a plant by locating areas with high frequency and logical relationships close to each other. It provides layout design guidelines for factory, product systems, hospitals, school, etc. in practice over the past few decades [16, 33]. There are some general steps for designing the layout in SLP:

- Analysing the relationship between the various operating units, including the relationship of logistics and nonlogistics.
- Obtaining the comprehensive relationship of the operating units.
- Determining the distance between each operating unit, according to the close relationship in a relationship chart. Then, arranging the position of each operating unit and drawing the position diagram.
- Combining the actual space of each operating unit with the position diagram to form the area relationship diagram of operating units.
- Correcting and adjusting the area relationship diagram to obtain several feasible layout schemes.
- Finally, evaluating the layout schemes by multifactor weighted analysis to obtain the optimal layout scheme.

**Figure 1:**
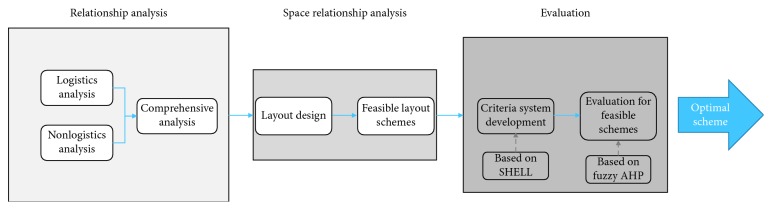
The structure of the proposed method.

In general, there are three main steps in SLP analysis, relationship diagram, and space relationship diagram and evaluation. The flowchart for SLP for an operating theatre proposed in this paper is shown in following Figure 1.

The relationship diagram includes the logistics, nonlogistics, and comprehensive relationships; it needs to start with an in-depth discussion of work relationships. In the manufacturing/service systems, each unit is related to every other unit, and it can be described by closeness ratings, which use the letters to represent the importance of closeness for each department pair. The closeness rating is described in Table 1 [16], with letter A being the most important and *X* being an undesirable pairing.

The closeness ratings are able to deal with the situation, which involves multiple criteria and can allow for subjective input from analysis or managers to indicate the relative importance of each combination of department pairs. After the relationship analysis, the area of each unit should be considered to draw the relative position relationship diagram and then to design the spatial relationship diagram to obtain the feasible layout schemes.

### 2.2. Human Reliability Analysis

Human reliability analysis relates to methodologies for anticipating and identifying the errors and weaknesses in the systems and assessing the effect of those failures that relate to human action or inaction [34]. It is a useful tool to design the objects, facilities, and environments that people use by analysis of the relevant information about human characteristics and behavior [35]. There are many tools, which are derived by different people in different industries for different purposes to analyze human reliability in systems. In this paper, the SHELL model (named after the initial letters of the five components' names, i.e., software, hardware, environment, liveware, and central liveware) is proposed to analyze the human reliability.

The SHELL model is a method which is used to describe the behavior and relationship of interactive systems regarding the human factors issues [26]. In the SHELL model, humans are considered as an integrated, important, and inseparable component of the production and service systems. Therefore, humans are centred in the model and connected with other components. In this model, the interfaces between the centre person and the other four components (hardware, software, environment, and liveware) are analyzed carefully, and it emphasized the interfaces rather than the components.

**Figure 2:**
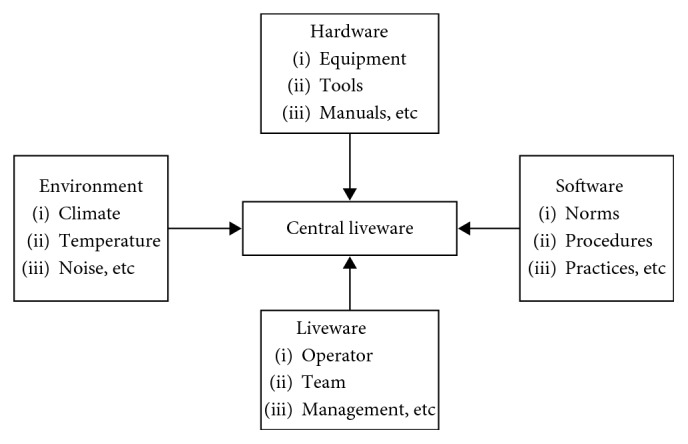
The SHELL model.

The five elements of the SHELL model (Figure 2) are introduced as follows [36]:

- Hardware is the element that shows the physical and nonhuman factors of the whole system, such as operator equipment, vehicles, and tools.
- Software represents all nonphysical resources, intangible aspects of systems, for organic operations, like organizational policies/rules, procedures, and norms.
- Environment includes the internal and external environments. The internal environment represents the factors that influence the working location, like climate, temperature, vibration, and noise, and the external environment represents the social-political and economic factors.
- Liveware represents the human element, and it considers about the teamwork, communication, leadership, etc.
- Central liveware is also the liveware, and it is regarded as the core element of the SHELL model.

### 2.3. Fuzzy Analysis Hierarchy Process

Analytic hierarchy process (AHP) is a multiattribute decision-making methods which was developed by Saaty and has been extensively studied and refined [37]. It is one of the most convenient methodologies for organizing and analyzing complex decisions and for dealing with complex problems where both qualitative and quantitative aspects need to be considered [28]. However, there are some weaknesses in AHP. For instance, it does not consider the ambiguity associated with the judgment of decision-makers regarding numeric values [38]. Fuzzy theory is considered to address this problem; it allows decision-makers to express approximate or flexible preferences using fuzzy numbers [39]. Fuzzy AHP is able to deal with imprecision and subjectivity in the pair-wise comparison process. Chang [40, 41] has introduced a new approach to handle fuzzy AHP with the use of an triangular fuzzy number and the extent analysis method. He introduced synthetic extent value *S*_*i*_ of the pair-wise comparison using the extent analysis method. It is concluded that the extent analysis method can deal with the time complexity better. However, it did not solve very well in the space complexity. Moreover, when the model is applied in the complex decision problem, it is difficult to get the value of fuzzy synthetic extent sometimes due to the complexity of the object and goal set.

When the decision-makers face a complex and uncertain problem and express their comparison judgments as uncertain ratios, the fuzzy sets and fuzzy numbers can be used to cope with such uncertain judgments. The triangular fuzzy number is used in fuzzy AHP decision-making model. A triangular fuzzy number $\tilde{\text{N}}$ is defined by three real numbers *a* ≤ *b* ≤ *c* and is characterized by a linear piecewise continuous membership function $\mu_{\tilde{\text{N}}}\left(x\right)$ of the type:

(1) $$\begin{array}{c} \mu_{\tilde{\text{N}}}\left(x\right)=\left\{\begin{array}{cc} \frac{x-a}{b-a}, & a\leq x\leq b,\\ \frac{c-x}{c-b}, & b\leq x\leq c,\\ 0, & \text{otherwise}. \end{array}\right. \end{array}$$

The fuzzy number $\tilde{\text{N}}$ is often expressed as a triple (*a, b, c*), where *a*, *b*, and *c* are the lower, the mean, and the upper bounds, respectively.

Let the fuzzy triangular numbers be described as ${\tilde{\text{a}}}_{ij}=\left(l_{ij},m_{ij},u_{ij}\right)$.

As in the traditional AHP, a fuzzy reciprocal comparison matrix $\tilde{\text{A}}=\left\{{\tilde{\text{a}}}_{ij}\right\}$ is constructed as

(2) $$\begin{array}{c} \tilde{\text{A}}=\left[\begin{array}{ccc} \begin{array}{c} \begin{array}{cc} 1 & {\tilde{\text{a}}}_{12} \end{array}\\ \begin{array}{cc} {\tilde{\text{a}}}_{21} & 1 \end{array} \end{array} & \begin{array}{c} \cdots \\ \ldots \end{array} & \begin{array}{c} {\tilde{\text{a}}}_{1n}\\ {\tilde{\text{a}}}_{2n} \end{array}\\ \begin{array}{cc} \vdots & \vdots \end{array} & \ddots & \vdots \\ \begin{array}{cc} {\tilde{\text{a}}}_{n1} & {\tilde{\text{a}}}_{n2} \end{array} & \cdots & 1 \end{array}\right], \end{array}$$

where ${\tilde{\text{a}}}_{ji}=1/{\tilde{\text{a}}}_{ij}$. For the complex systems, it needs to assess the alternatives by more than one decision-maker. In order to consider multiple decision-makers, Van Laarhoven and Pedrycz [42] constructed the fuzzy reciprocal matrix as follows:

(3) $$\begin{array}{c} \tilde{\text{A}}=\left[\begin{array}{cccc} & {\tilde{\text{a}}}_{121} & & {\tilde{\text{a}}}_{1n1}\\ & {\tilde{\text{a}}}_{122} & & {\tilde{\text{a}}}_{1n2}\\ 1 & \vdots & \ldots & \vdots \\ & {\tilde{\text{a}}}_{12{\text{P}}_{12}} & & {\tilde{\text{a}}}_{1n{\text{P}}_{1n}}\\ {\tilde{\text{a}}}_{211} & & & {\tilde{\text{a}}}_{2n1}\\ {\tilde{\text{a}}}_{212} & & & {\tilde{\text{a}}}_{2n2}\\ \vdots & 1 & \ldots & \vdots \\ {\tilde{\text{a}}}_{21{\text{P}}_{21}} & & & {\tilde{\text{a}}}_{2n{\text{P}}_{2n}}\\ \vdots & \vdots & & \\ {\tilde{\text{a}}}_{n11} & {\tilde{\text{a}}}_{n21} & & \\ {\tilde{\text{a}}}_{n12} & {\tilde{\text{a}}}_{n22} & \ddots & \vdots \\ \vdots & \vdots & \ldots & 1\\ {\tilde{\text{a}}}_{n1{\text{P}}_{n1}} & {\tilde{\text{a}}}_{n2{\text{P}}_{n2}} & & \end{array}\right], \end{array}$$

where $\tilde{\text{A}}=\left\{{\tilde{\text{a}}}_{ij{\text{P}}_{ij}}\right\}$ and ${\tilde{\text{a}}}_{ij{\text{P}}_{ij}}$ are fuzzy ratios assessed by multiple decision-makers and ${\tilde{\text{a}}}_{ji{\text{P}}_{ji}}=1/{\tilde{\text{a}}}_{ij{\text{P}}_{ij}}$.

(4) $$\begin{array}{c} l_{ij}=\left(\frac{\sum l_{ij{\text{P}}_{ij}}}{m}\right),\\ m_{ij}=\left(\frac{\sum m_{ij{\text{P}}_{ij}}}{m}\right),\\ l_{ij}=\left(\frac{\sum u_{ij{\text{P}}_{ij}}}{m}\right). \end{array}$$

where *m* is the number of decision-makers. After constructing the comparison matrix, the fuzzy weight should be calculated as follows:

(5) $$\begin{array}{c} {\text{D}}_{i}^{k}=\frac{\overset{n}{\underset{j=1}{\sum }}{\text{a}}_{ij}^{k}}{\left(\overset{n}{\underset{i=1}{\sum }}\overset{n}{\underset{j=1}{\sum }}{\text{a}}_{ij}^{k}\right)},\;\text{i}=1,2,\ldots ,n. \end{array}$$

If M_1_(*l*_1_, *m*_1_, *u*_1_) and M_2_(*l*_2_, *m*_2_, *u*_2_) are the fuzzy triangular numbers, then

(6) $$\begin{array}{c} v\left({\text{M}}_{1}\geq {\text{M}}_{2}\right)={\text{sup}}_{x\geq y}\left[\min\left(u_{{\text{M}}_{1}}\left(x\right),u_{{\text{M}}_{2}}\left(y\right)\right)\right], \end{array}$$

(7) $$\begin{array}{c} v\left({\text{M}}_{1}\geq {\text{M}}_{2}\right)=u\left(\text{d}\right)=\;\;\left\{\begin{array}{cc} 1, & m_{1}\geq m_{2},\\ \frac{l_{2}-u_{1}}{\left(m_{1}-u_{1}\right)-\left(m_{2}-l_{2}\right)}m_{1}\leq m_{2}, & u_{1}\geq l_{2},\\ 0, & \text{otherwise}. \end{array}\right. \end{array}$$

(8) $$\begin{array}{c} v\left(\text{M}\geq {\text{M}}_{1},{\text{M}}_{2},\ldots ,{\text{M}}_{k}\right)=\text{min}\;\;v\left(\text{M}\geq {\text{M}}_{i}\right),\;i=1,2,\ldots ,k. \end{array}$$

Then, calculate the fuzzy utility for an alternative:

(9) $$\begin{array}{c} {\text{TV}}_{{\text{A}}_{i}}=\overset{\text{h}}{\underset{i=1}{\sum }}\left({\text{V}}_{i}\times {\text{TW}}_{\text{I}}\right). \end{array}$$

## 3. Results and Discussion

The application of the proposed method was undertaken in a hospital, which wants to redesign the layout of the operating theatre. The application steps are shown as follows:

- Design of the layout plans for an operating theatre. In this step, this paper uses the SLP method to design the operating theatre layout. It analyzes the logistics and nonlogistics relationships among the different units in an operating theatre and designs the layout plans.
- Development of the criteria for evaluation. Considering a human error and using the SHELL model from the four interfaces of the human reliability analysis to develop the criteria system.
- Evaluation of the layout schemes using fuzzy AHP. In the last step, this paper applies the fuzzy AHP to deal with imprecision and subjectivity in the pair-wise comparison process to assess the layout plans and then choose the optimal one.

### 3.1. Design of the Operating Theatre Layout Schemes

The hospital is located in Shanghai. It is a general hospital with 9 operating rooms in the operating theatre. There are eight operation units in the operating theatre, i.e., two staff rest rooms (RR), one preoperative holding units (PHU), one postanesthesia care unit (PACU), one nursing station (NS), one equipment room (ER), nine operating rooms (OR), one sterile goods storage (SGS), and one central sterile supply department (CSSD).

SLP is applied to reallocate operating theatre spaces and services. The logistics analysis means the real movement of people and goods. It is determined by the move sequence and the amount of movement of people and goods. The nonlogistics represents the mutual relation among the units, and it consists of the analysis of relation of process and contact of the product and personnel. There are many influencing factors in the interrelation of units in operating theatre layout planning, e.g., convenience of supervision and management, safety and pollution, and frequency of the medical personnel contact [16]. Finally, we consider the relative importance of the logistics and nonlogistics as 1 : 1. Combine the logistics and nonlogistics relationships, and calculate the comprehensive relationship. According to the field research and the expert evaluation, the comprehensive relationship diagram is obtained (Table 2).

**Figure 3:**
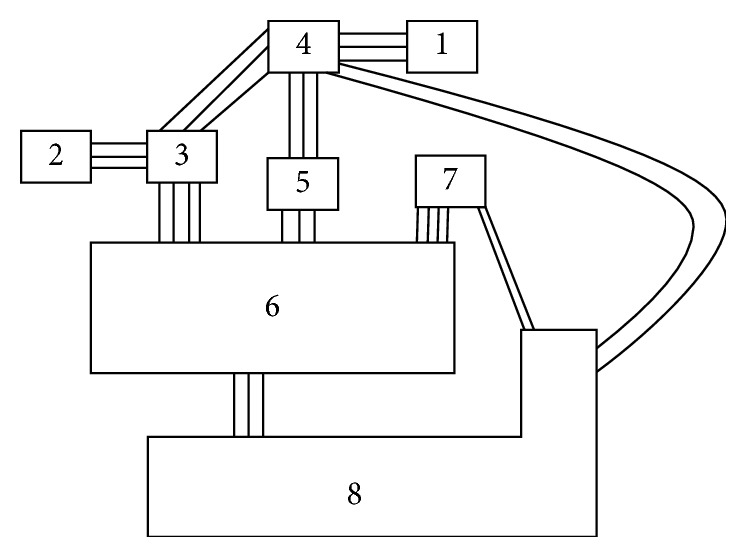
The relative position relationship diagram. 1, Staffing rest rooms; 2, PHU; 3, PACU; 4, nursing stations; 5, equipment room; 6, operating rooms; 7, sterile goods storage; 8, CSSD.

After the analysis of the relationship of each unit, the relative position relationship diagram is drawn by considering the area of each unit (Figure 3). Here, the space of each operation unit and its shape are not directly considered. Instead, the relative position between the units is arranged from the close relationship.

**Figure 4:**
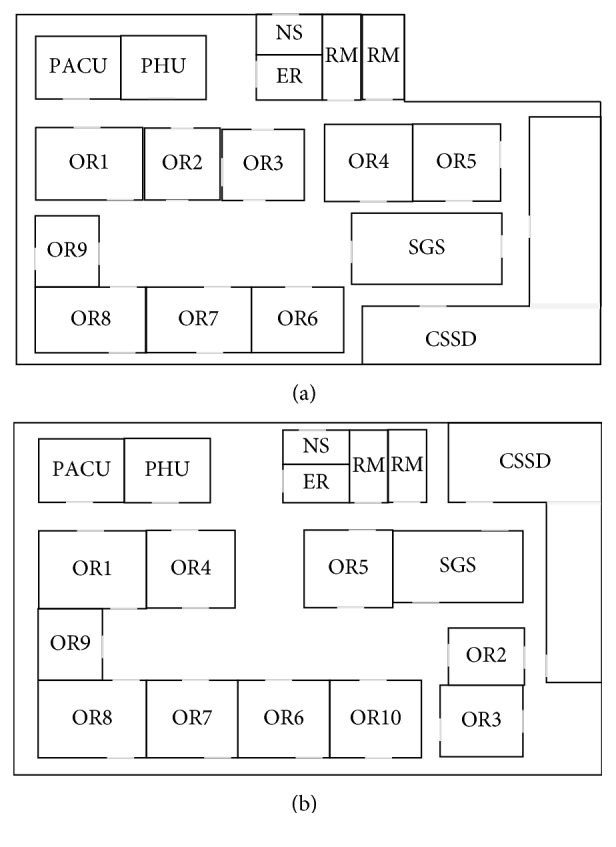
The two layout plans for the OT: (a) scheme A1; (b) scheme A2.

Finally, the area of each unit should be considered to design the spatial relationship diagram. According to the relative position relationship analysis and field research, the spatial layout schemes are drawn as below (Figure 4).

### 3.2. Development of the Criteria System

**Figure 5:**
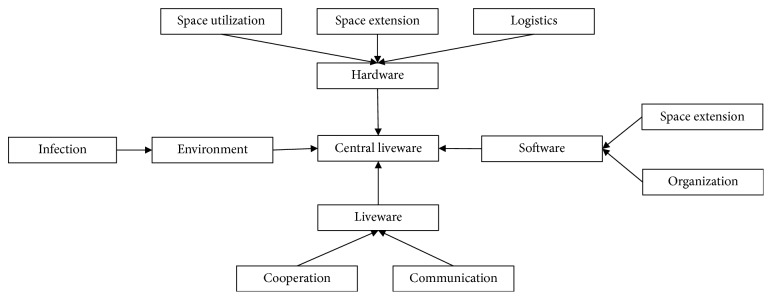
The SHELL model for development of criteria.

According to the introduction of the SHELL model, the four interfaces of the human reliability analysis are defined: L-H, L-S, L-E, and L-L. The operating theatre nurses are the most important components in the operating theatre. Therefore, this paper names the nurses in an operating theatre as the central liveware. The SHELL model for human reliability of the layout is given in Figure 5. In the L-H subsystem, this paper focuses on the space utilization, space extension, and logistics. In the L-S subsystem, it focuses on the procedure and organization. In the L-E subsystem, it studies the infections. And lastly, in the L-L subsystem, it focuses on the cooperation and communication. Based on these components, the criteria system for evaluation is proposed.

### 3.3. Application of Fuzzy AHP

The facility layout design for an operating theatre is a complex and uncertain problem, as the imperfect information, ambiguous data, and uncertain factors. In this situation, it should consider the multiple attributes that are both quantitative and qualitative. The fuzzy AHP is used to tackle the uncertainty and imprecision of the evaluation process.

**Figure 6:**
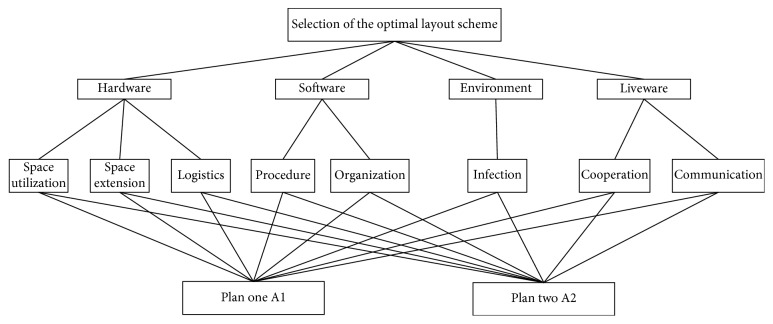
Decision hierarchy.

The fuzzy AHP decision problem is structured hierarchically at different levels. Each level consists of a number of decision elements which are divided into goals, criteria, and alternatives. The top level represents the overall goal, while the lowest level is composed of the possible alternatives. This paper takes the overall goal as selecting the best layout scheme. And the possible alternatives are the two layout schemes we have obtained. Between the top and lowest levels, there are some intermediate levels, which embody the decision criteria and subcriteria. Using the SHELL, we consider the four interfaces as the four criteria and the elements in each interface as the subcriteria. The hierarchy construction of fuzzy AHP for selecting the best layout scheme for the operating theatre is described in Figure 6.

The relative importance of the different decision elements (weights of criteria and scores of alternatives) is evaluated indirectly from comparison judgments. The decision-makers are required to provide their preferences by comparing all criteria, subcriteria, and alternatives with respect to upper level decision elements.

In order to assess the two layout schemes, we construct the assessment group: one head nurse, one surgeon, and one hospital administrator. The head nurse in the operating theatre is charge of the management and scheduling of operations. She knows the workflow of the operating theatre well. And she is familiar with the factors which influence the productivity of the operating theatre. The surgeon is the person who performs the operations for patients in the operating theatre. The efficiency and productivity of the operating theatre will impact on the operation time and then on satisfaction of patients. The hospital administrator should not only focus on the efficiency and effectiveness of the hospital operation but also consider the hospital's development and its future. The administrator must ensure an optimum functioning of the system components. These three decision-makers were asked to rate the level of the importance of the criteria. Their opinions about the relative importance of a pair of the first level of attributes are shown in Table 3.

Integrate the three experts' opinions and obtain the fuzzy matrix:

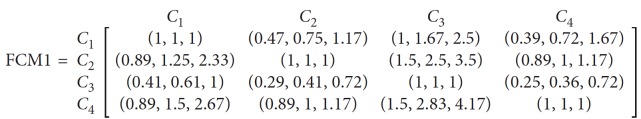
(10)


According to Equation (5), we can get the fuzzy weight of C1 as follows:

(11) $$\begin{array}{c} \overset{4}{\underset{i=1}{\sum }}\overset{4}{\underset{j=1}{\sum }}{\text{a}}_{ij}=\left(1,1,1\right)+\left(0.47,0.75,1.17\right)+\ldots +\left(1,1,1\right)=\left(13.37,18.6,26.79\right),\\ \overset{4}{\underset{j=1}{\sum }}{\text{a}}_{1j}=\left(1,1,1\right)+\left(0.47,0.75,1.17\right)+\left(1,1.67,2.5\right)+\left(0.39,0.72,1.67\right)=\left(2.86,4.14,6.34\right),\\ {\text{D}}_{\text{C}1}=\frac{\overset{4}{\underset{j=1}{\sum }}{\text{a}}_{1j}}{\overset{4}{\underset{i=1}{\sum }}\overset{4}{\underset{j=1}{\sum }}}{\text{a}}_{ij}=\left(0.107,0.223,0.474\right). \end{array}$$

Similarly, we can get the fuzzy weights of C2, C3, and C4 as follows:

(12) $$\begin{array}{c} \text{DC}2=\left(0.149,0.309,0.598\right),\\ \text{DC}3=\left(0.073,0.128,0.257\right),\\ \text{DC}4=\left(0.160,0.287,0.674\right). \end{array}$$

Then, defuzzification is done for DC1, DC2, DC3, and DC4 to obtain the weight of d(C1), d(C2), d(C3), and d(C4) according to Equations (7) and (8):

(13) $$\begin{array}{c} \text{v}\left({\text{D}}_{\text{C}1}\geq {\text{D}}_{\text{C}2}\right)=\frac{0.149-0.474}{\left(0.223-0.474\right)-\left(0.309-0.149\right)}=0.791,\\ \text{v}\left({\text{D}}_{\text{C}1}\geq {\text{D}}_{\text{C}3}\right)=1,\\ \text{v}\left({\text{D}}_{\text{C}1}\geq {\text{D}}_{\text{C}4}\right)=\frac{0.160-0.474}{\left(0.223-0.474\right)-\left(0.287-0.160\right)}=0.831,\\ \text{d}\left(\text{C}1\right)=\text{min v}\left(\text{DC}1\geq \text{DC}2,\text{DC}3,\text{DC}4\right)=\text{min}\left(\text{0}.791,1,0.831\right)=.791,\\ \text{d}\left(\text{C}2\right)=\text{min v}\left(\text{DC}2\geq \text{DC}1,\text{DC}3,\text{DC}4\right)=\text{min}\left(1,1,1\right)=1,\\ \text{d}\left(\text{C}3\right)=\text{min v}\left(\text{DC}3\geq \text{DC}1,\text{DC}2,\text{DC}4\right)=\text{min}\left(\text{0}.612,0.374,0.379\right)=0.374,\\ \text{d}\left(\text{C}4\right)=\text{min v}\left(\text{DC}4\geq \text{DC}1,\text{DC}2,\text{DC}3\right)=\text{min}\left(1,0.960.1\right)=0.960. \end{array}$$

Standardize the weight of d(C1), d(C2), d(C3), and d(C4):

(14) $$\begin{array}{c} \left(\text{WC}1,\text{WC}2,\text{WC}3,\text{WC}4\right)=\left(\text{0}.2531,0.3200,0.1197,0.3072\right). \end{array}$$

After getting the first level criteria weights, we should then calculate the second level criteria weights. The evaluation team compared the attributes with respect to the corresponding criteria. The evaluation team's opinions about the relative importance of a pair of attributes are shown in Tables 4 [Table tab5]–6.

Integrate the three experts' opinions and obtain the fuzzy matrix:

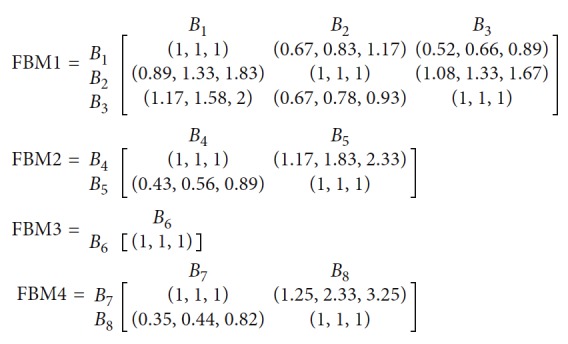
(15)


Similarly, according to Equations (5), (7), and (8), we calculate the fuzzy weight of B1–B8 and defuzzificate them to obtain the weights and standardize the weights. Therefore, the standardization of the B1–B8's weights is shown as follows:

(16) $$\begin{array}{c} \left(\text{WB}1,\text{WB}2,\text{WB}3\right)=\left(\text{0}.2115,0.4196,0.3689\right),\\ \left(\text{WB}4,\text{WB}5\right)=\left(\text{0}.7854,0.2146\right),\\ \left(\text{WB}6\right)=1,\\ \left(\text{WB}7,\text{WB}8\right)=\left(\text{0}.7973,\text{0}.2027\right). \end{array}$$

Considering the upper level criteria weights and calculation of the weights of lower level criteria (subcriteria), the results (the weights of B1–B8) are shown in Table 7.

After calculation of the weights of the level B, the weights of alternatives (the layout plans) should be calculated. The experts compared the two schemes with respect to each attribute. After assigning the weights to each attribute, the experts compared both alternatives: A1 and A2. The experts' opinions about the relative importance of a pair of attributes are shown in Tables 8 [Table tab9] [Table tab10] [Table tab11] [Table tab12] [Table tab13] [Table tab14]–15. Finally, on adding the weights for layout plan alternatives multiplied by the weights of the corresponding criteria, a final score is obtained for each alternative.

Integrate the three experts' opinions and obtain the fuzzy matrix:

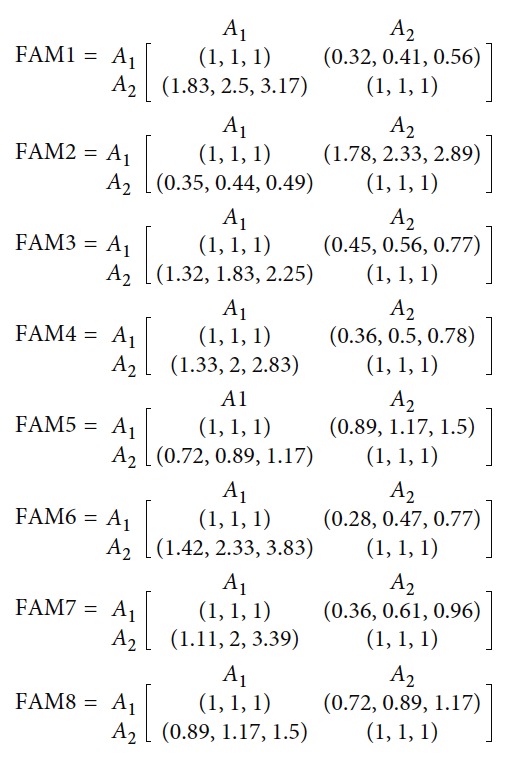
(17)


According to Equations (5), (7), and (8), we calculate the fuzzy weight of alternatives (plans) and defuzzificate them to obtain the weights and then standardize the weights. According to Equation (9), we can obtain the total weights of the two alternatives. The results are shown in Table 16.

From the results in Table 16, it shows that the weight of A2 is 0.694, and it is higher than A1. It means that A2 is the better choice than A1. Therefore, A2 is the optimal layout of the operating theatre.

## 4. Discussion

The design of hospital facilities can have a large impact on efficiency and outcomes, especially the design of the facility for the operating theatre. The efficient design of space can have a large impact on the efficiency and effectiveness of the operating theatre process. In this paper, we present an approach that utilizes human reliability analysis and fuzzy AHP in selecting layout candidate schemes. We also propose a hierarchy criteria model to incorporate information from different factors by using SHELL technologies. It offers a reference for future layout plan selection. Because this study takes into account the criteria of different factors, the process by which the model selects layout plans is appropriate. It is clear that assignment of criteria weights is based on the experience of the experts involved in the weight assignment process. A weight is assigned as objectively as possible by applying techniques like the fuzzy AHP. In the final aggregation process, factor weights are evaluated by all factors, as they all play a very important role in selecting a layout plan.

Hospitals are complex service environments in which patients, nurses, and surgeons are all parts of the system. The design of facilities in operating theatres is more complicated because there are multiple factors that interact and affect the efficiency of the operating theatre process. Numerous literature and studies have applied SLP to obtain the most effective facility arrangement and then minimize the material handling costs. In this article, SLP provides a framework for designing the facility layout plans, taking account of the logistics and nonlogistics relationships. Hospitals are not factories; they are sociotechnical systems. It requires more scientific methods for design and optimization. It also needs to consider more corresponding factors when making a decision for layout plans. Some researchers consider only the logistics efficiency and space utilization as the criteria for selecting the optimal layout plan [16]. However, it is not enough to consider only these two criteria. In health care systems, it should consider more factors like the human factors, the human errors, and system safety. HRA provides a framework for developing the assessment systems to predict the occurrence of “human errors” of systems. It has been used in high risk industries to prevent accidents, the consequences of which would be catastrophic. It aims at reducing errors such that the risk of the system is as low as possible. In order to develop the comprehensive criteria for selecting the best layout plans of an operating theatre, this article uses SHELL to develop the criteria system for making decisions. The case study illustrates the process of identifying the optimal layout. The candidate plans based on the criteria system are aggregated based on their weights. The method is very practical for health care systems planning. In the end, the best layout plan is achieved, and it can be taken as the optimal layout plan for an operating theatre.

## 5. Conclusions

The increasing demand for health care is one of the greatest challenges faced by governmental authorities not only in developing countries but also in developed countries, due to aging populations, new technologies, and life style factors. The development of our study is motivated by the desire to search for a scientific method for the facility layout in hospitals, to increase the efficiency and effectiveness of hospital operations. We have integrated HRA and a multicriteria evaluation technique, together with fuzzy AHP, to select an optimal layout plan for operating theatres. The SHELL model is used to develop the criteria system from the four factors: hardware, software, environment, and liveware, including eight criteria categorized in four factors. The criteria were determined and then compared according to their importance. Fuzzy AHP offered an objective weight assignment process. Furthermore, the use of the set of weights according to their importance provided great flexibility in the aggregation procedure. The proposed methodology was applied successfully to a hospital in Shanghai as a real case study. The methodology also gives some suggestions about successful layout plan design. The proposed methodology is flexible and can be used for other sectors, with some appropriate changes. Humans are often uncertain when giving the precise evaluation scores. However, fuzzy AHP can overcome this difficulty. Fuzzy AHP is able to convert the subjective cognition into an information entity with the expert system. The expert system can be used before and after the layout is selected. The lessons from this case or other applications can be added into the knowledge base of the expert system.

The design of the facility layout for an operating theatre is difficult and complex; it needs more scientific methods and theories for design and optimization. In this study, we propose a new methodology with fuzzy AHP and human reliability assessment, to design and select the best layout plans for an operating theatre. It provides a new theoretical framework for hospital administration to design facility layout in hospitals. There are numerous strengths in this study. However, for future study, the following topics can be considered: (i) the fuzzy scores and the importance of the criteria can be obtained by involving more participants with different expertise and knowledge; (ii) to adapt the methodology to different hospital layout designs or for use in other public sectors.

## Figures and Tables

**Table 1:** The closeness rating.

Code	Degree of closeness	Proportion of logistics quantity (%)	Value	Line code
A	Absolutely necessary	40	4	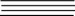
E	Especially important	30	3	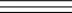
I	Important	20	2	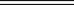
O	Ordinary closeness	10	1	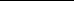
U	Unnecessary	0	0	
X	Undesirable	—	−1	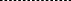

**Table 2:** The comprehensive relationship among different units in the operating theatre.

Unit	RR	PHU	PACU	NS	ER	OR	SGS	CSSD
RR		U	U	E	U	I	U	U
PHU	U		E	E	U	E	U	U
PACU	U	E		E	U	A	U	U
NS	E	E	E		E	E	E	I
ER	U	U	U	E		E	U	U
OR	I	E	A	E	I		E	E
SGS	U	U	U	E	U	A		U
CSSD	U	U	U	I	U	O	I	
Closeness	8	16	18	34	9	37	12	7
Reorder	7	4	3	2	6	1	5	8

**Table 3:** Pair-wise comparisons of main attributes C.

Criteria	C1	C2	C3	C4
C1	(1, 1, 1)	(1/3, 2/3, 1) (2/3, 1, 3/2) (2/5, 4/5, 1)	(1, 2, 3) (1, 3/2, 2) (1, 3/2, 5/2)	(1/3, 2/3, 1) (1/2, 1, 3/2) (1/3, 1/2, 1)

C2	(1, 3/2, 3) (2/3, 1, 3/2) (1, 5/4, 5/2)	(1, 1, 1)	(1, 2, 4) (3/2, 5/2, 3) (2, 3, 7/2)	(1, 1, 1) (2/3, 1, 3/2) (1, 1, 1)

C3	(1/3, 1/2, 1) (1/2, 2/3, 1) (2/5, 2/3, 1)	(1/4, 1/2, 1) (1/3, 2/5, 2/3) (2/7, 1/3, 1/2)	(1, 1, 1)	(1/5, 1/3, 1) (1/4, 1/3, 1/2) (2/7, 2/5, 2/3)

C4	(1, 3/2, 3) (2/3, 1, 2) (1, 2, 3)	(1, 1, 1) (2/3, 1, 3/2) (1, 1, 1)	(1, 3, 5) (2, 3, 4) (3/2, 5/2, 7/2)	(1, 1, 1)

**Table 4:** Pair-wise comparisons of attributes B with respect to hardware C1.

Criteria	B1	B2	B3
B1	(1, 1, 1)	(1, 1, 1) (1/3, 1/2, 1) (2/3, 1, 3/2)	(2/5, 1/2, 2/3) (1/2, 2/3, 1) (2/3, 4/5, 1)

B2	(1, 1, 1) (1, 2, 3) (2/3, 1, 3/2)	(1, 1, 1)	(1, 1, 1) (1, 3/2, 2) (5/4, 3/2, 2)

B3	(3/2, 2, 5/2) (1, 3/2, 2) (1, 5/4, 3/2)	(1, 1, 1) (1/2, 2/3, 1) (1/2, 2/3, 4/5)	(1, 1, 1)

**Table 5 tab5:** Pair-wise comparisons of attributes B with respect to hardware C2.

Criteria	B4	B5
B4	(1, 1, 1)	(1, 2, 5/2) (1, 3/2, 2) (3/2, 2, 5/2)

B5	(2/5, 1/2, 1) (1/2, 2/3, 1) (2/5, 1/2, 2/3)	(1, 1, 1)

**Table 6:** Pair-wise comparisons of attributes B with respect to hardware C4.

Criteria	B7	B8
B7	(1, 1, 1)	(1, 3, 5) (3/2, 2, 5/2) (5/4, 2, 9/4)

B8	(1/5, 1/3, 1) (2/5, 1/2, 2/3) (4/9, 1/2, 4/5)	(1, 1, 1)

**Table 7:** The weights of the level B.

Criteria	C1	C2	C3	C4	Weights of level B
	0.2531	0.3200	0.1197	0.3072	
B1	0.2115				0.0535
B2	0.4196				0.1062
B3	0.3689				0.0934
B4		0.7854			0.2513
B5		0.2146			0.0687
B6			1		0.1197
B7				0.7973	0.2449
B8				0.2027	0.0623

**Table 8:** Pair-wise comparisons of plan with respect to space utilization B1.

Scheme	A1	A2
A1	(1, 1, 1)	(2/5, 1/2, 2/3) (2/7, 1/3, 1/2) (2/7, 2/5, 1/2)

A2	(3/2, 2, 5/2) (2, 3, 7/2) (2, 5/2, 7/2)	(1, 1, 1)

**Table 9 tab9:** Pair-wise comparisons of plan with respect to space extension B2.

Scheme	A1	A2
A1	(1, 1, 1)	(4/3, 2, 8/3) (5/2, 3, 7/2) (3/2, 2, 5/2)

A2	(3/8, 1/2, 3/4) (2/7, 1/3, 2/5) (2/5, 1/2, 2/3)	(1, 1, 1)

**Table 10 tab10:** Pair-wise comparisons of plan with respect to logistics B3.

Scheme	A1	A2
A1	(1, 1, 1)	(1/2, 2/3, 5/6) (4/9, 1/2, 4/5) (2/5, 1/2, 2/3)

A2	(6/5, 3/2, 2) (5/4, 2, 9/4) (3/2, 2, 5/2)	(1, 1, 1)

**Table 11 tab11:** Pair-wise comparisons of plan with respect to procedure B4.

Scheme	A1	A2
A1	(1, 1, 1)	(1/3, 1/2, 1) (2/5, 1/2, 2/3) (1/3, 1/2, 2/3)

A2	(1, 2, 3) (3/2, 2, 5/2) (3/2, 2, 3)	(1, 1, 1)

**Table 12 tab12:** Pair-wise comparisons of plan with respect to organization B5.

Scheme	A1	A2
A1	(1, 1, 1)	(1, 1, 1) (1, 3/2, 2) (2/3, 1, 3/2)

A2	(1, 1, 1) (1/2, 2/3, 1) (2/3, 1, 3/2)	(1, 1, 1)

**Table 13 tab13:** Pair-wise comparisons of plan with respect to infection B6.

Scheme	A1	A2
A1	(1, 1, 1)	(1/4, 1/3, 1/2) (2/5, 2/3, 1) (1/5, 2/5, 4/5)

A2	(2, 3, 4) (1, 3/2, 5/2) (5/4, 5/2, 5)	(1, 1, 1)

**Table 14 tab14:** Pair-wise comparisons of plan with respect to cooperation B7.

Scheme	A1	A2
A1	(1, 1, 1)	(1/5, 1/3, 1) (2/7, 1/2, 2/3) (3/5, 1, 6/5)

A2	(1, 3, 5) (3/2, 2, 7/2) (5/6, 1, 5/3)	(1, 1, 1)

**Table 15:** Pair-wise comparisons of plan with respect to communication B8.

Scheme	A1	A2
A1	(1, 1, 1)	(1, 1, 1) (1/2, 2/3, 1) (2/3, 1, 3/2)

A2	(1, 1, 1) (1, 3/2, 2) (2/3, 1, 3/2)	(1, 1, 1)

**Table 16:** The weights of the two plans.

Scheme	B1	B2	B3	B4	B5	B6	B7	B8	Total weights
	0.0535	0.1062	0.0934	0.2513	0.0687	0.1197	0.2449	0.0623	
A1	0	1	0.0180	0.1435	0.5731	0.1829	0.3033	0.4253	0.306
A2	1	0	0.9820	0.8565	0.4269	0.8171	0.6967	0.5747	0.694

WA1 = 0.0535 *∗* 0 + 0.1062 *∗* 1 + 0.0934 *∗* 0.0180 + 0.2513 *∗* 0.1435 + 0.0687 *∗* 0.5731 + 0.1197 *∗* 0.1829 + 0.2449 *∗* 0.3033 + 0.0623 *∗*0.4253 = 0.306. WA2 = 0.0535*∗*1 + 0.1062*∗*0 + 0.0934*∗*0.9820 + 0.2513*∗*0.8565 + 0.0687 + 0.4269 + 0.1197*∗*0.8171 + 0.2449*∗*0.6967 + 0.0623*∗*0.5747 = 0.694.

## Data Availability

The data used to support the findings of this study are included within the article.
